# Context Matters in Evidence Implementation Globally: Comment on "Stakeholder Perspectives of Attributes and Features of Context Relevant to Knowledge Translation in Health Settings: A Multi-Country Analysis"

**DOI:** 10.34172/ijhpm.2021.179

**Published:** 2021-12-27

**Authors:** Marie-Pierre Gagnon

**Affiliations:** Faculty of Nursing Sciences, Université Laval, Quebec City, QC, Canada.

**Keywords:** Knowledge Translation, Context, Implementation

## Abstract

Context influences the effectiveness of healthcare interventions and should be considered to inform their implementation. However, context remains poorly defined in the knowledge translation (KT) literature. The paper by Squires and colleagues constitutes a valuable contribution to the field of KT as it provides the basis for a comprehensive framework to assess the influence of context on implementation success. In their study, Squires et al identified 66 context features, grouped into 16 attributes. Their findings highlight a great convergence in the context features mentioned by stakeholders across countries, experience levels and roles in KT. Thus, the proposed framework could eventually transfer to several implementation settings. However, all study participants were from high-income countries. It would therefore be important to replicate this research in low- and middle-income countries (LMICs). A common understanding of what context means is essential to assessing its influence on the implementation of healthcare interventions globally.

## Introduction

 The challenge of implementing evidence-informed interventions in healthcare has been the focus of research for over two decades. It is widely recognized that the context in which an intervention is implemented is key to understand the success or failure of implementation efforts.^1^ However, context remains poorly defined in the knowledge translation (KT) and implementation science (IS) literature. Also, the difficulty to account for all aspects of the context that could influence the success of healthcare interventions is a major issue.^2^

 Inconsistency in the way context has been conceptualized may call into question the importance it has been given in KT/IS research. In implementation frameworks, context often refers to the implementation setting or the physical environment in which healthcare practices occur.^3^ Other definitions include more dynamic aspects in assessing context such as networks and relationships.^4^

 The study by Squires and colleagues^5^ contributes to fill these gaps as it provides an overview of the contextual factors relevant to KT/IS research. A total of 66 unique context features, grouped into 16 context attributes, were identified through interviews with stakeholders in four countries who had tacit knowledge regarding the implementation of evidence-informed interventions. This classification provides a comprehensive list of contextual factors influencing KT in healthcare.

 This study also addresses three important issues. First, there is considerable inconsistency in terms used to define contextual determinants and how they are conceptualized in KT/IS frameworks.^6^ The contribution of Squires et al is to provide a better understanding of what the abstract concepts proposed in different implementation frameworks mean in practice.

 Second, given the lack of a consensual taxonomy and common classification system, the information reported on contextual factors in implementation studies of healthcare interventions is generally limited.^6^ The comprehensive list proposed by Squires et al could thus serve as a basis to develop a checklist for reporting contextual elements in implementation studies.

 Third, although several KT/IS frameworks include a contextual dimension, there is a lack of guidance on how to assess the relative influence of various context features on the outcomes of healthcare interventions. Thus, the contextual features and attributes identified by Squires et al^5^ could inform the development of a common assessment tool to measure the relative influence of specific factors in different contexts.

 The Squires et al paper^5^ is therefore most relevant to address these issues, but some aspects need to be considered in order to further advance knowledge in this area. This commentary reflects on the main contributions of the work by Squires and colleagues and points out potential avenues for further developments.

## Looking Into the Black Box

 In the last 20 years, several authors have proposed a conceptualization of context in the field of KT/IS. For instance, McCormack et al^3^ provided a conceptual analysis of context as reflected in the Promoting Action on Research Implementation in Health Services (PARiHS) framework. In the PARiHS framework, context is considered as one of the three cornerstones that influence evidence implementation, together with evidence and facilitation, and includes four sub-elements: receptive context, culture, leadership, and evaluation.^7^

 Some KT/IS frameworks make a distinction between determinants related to the internal context in which the intervention is implemented and those related to the broader context. For instance, the Consolidated Framework for Implementation Research distinguishes the inner setting, which concerns for instance the structural characteristics, the culture and the climate for implementation, and the outer setting that includes constructs considered outside the organization, such as patient needs and external policies and incentives.^8^

 Later, another checklist was developed by Flottorp and colleagues.^9^ The Tailored Implementation in Chronic Diseases checklist is based on a systematic review of KT determinants and a consensus process. Among the 7 broad domains included in the Tailored Implementation in Chronic Diseases, 5 are related to context: patient factors, professional interactions, incentives and resources, capacity for organizational change, and social, political and legal factors.

 A last example is provided by Tomoaia-Cotisel et al^10^ who propose a template to collect and report contextual factors at the practice, organization, and external environment levels. A total of 38 contextual factors were identified from a retrospective analysis of 14 implementation projects in primary care. These factors were grouped into 5 domains: the practice setting, the larger organization, the external environment, the implementation pathway, and the motivation for implementation. All the contextual factors identified in previous frameworks are found in the list developed by Squires and colleagues, providing support for its completeness.

 As Squires et al^5^ indicate, there is a lack of involvement of stakeholders responsible for implementing health interventions in defining the contextual factors to be considered. To address this gap, they interviewed 39 health system stakeholders from four countries (Australia, Canada, the United Kingdom, and the United States) to gather their views on the contextual features that influenced KT. These stakeholders were responsible for the design or implementation of interventions, programs and change processes, and included both change agents within the health system and KT researchers. Thus, the proposed classification is informed by tacit knowledge and should better reflect the perspective of those involved in the implementation of healthcare interventions.

 A common classification of contextual factors involved in the implementation of interventions can facilitate their reporting in a structured way, thus facilitating their use in meta-analyses or other types of knowledge synthesis. Specific reporting guidelines have been developed to provide guidance on how to report implementation studies. For instance, the revised *Criteria for Reporting the Development and Evaluation of Complex Interventions in healthcare* proposes 13 generic items to report information regarding the stages development, piloting, and evaluation of a complex intervention.^11^ Three of the items concern context more directly (description of internal facilitators and barriers; description of external conditions or factors which might have influenced the delivery of the intervention; and description of costs or required resources for the delivery of the intervention) and could be complemented with a checklist of specific contextual factors.

 Another example is the *Standards for Reporting Phase IV Implementation studies* (StaRI) that concerns the implementation of interventions supported by research findings into routine healthcare services.^12^ The StaRI includes 27 items to support researchers in describing both the implementation strategy and the effectiveness of the intervention. A detailed description of contextual factors based on the list developed by Squires et al^5^ could complement the description of some of the StaRI items such as the context in which the intervention was implemented, the characteristics of the targeted implementation sites and the population targeted by the intervention.

## When Context Is Complex

 For complex interventions involving a multiplicity of settings and stakeholders, context extends beyond the “physical” context related to the characteristics of the implementation sites to include networks and relationships. Thus, this is even more important to consider the context in which complex interventions are implemented in assessing their effectiveness.^1^ Documenting contextual factors involved in complex interventions as dynamic and interrelated networks would allow for a better understanding of the conditions that facilitate or limit KT. This represents a challenge for complex adaptive systems such as those involved in the implementation of healthcare interventions.^4^

 Recently, some frameworks that account for the complexity in KT/IS have been proposed. One of them is the Context and Implementation of Complex Interventions framework.^1^ The Context and Implementation of Complex Interventions framework includes three dimensions: context, implementation and setting. These dimensions are in interaction with each other and also interact with the intervention dimension. The context dimension comprises seven domains – geographical, epidemiological, socio-cultural, socio-economic, ethical, legal, and political – whereas the setting dimension refers to the specific physical location in which the intervention takes place. This dynamic relationship between intervention, implementation, context and setting can occur on a micro, meso and macro level.^1^

 For their part, Kitson et al^4^ have developed the KT Complexity Network model that combines the five key areas of the KT process (problem identification, knowledge creation, knowledge synthesis, implementation and evaluation) and the characteristics of Complex Adaptative Systems. This model allows for the consideration of the dynamic and complex nature of the KT process that is too often conceptualized as a linear or cyclic model. Using such framework would allow considering contextual elements within each sub-networks or clusters of the KT process as nonlinear, interconnected and adaptive.

 It is thus important to acknowledge the adaptive capacity of the implementation context when designing complex interventions.^2^ The framework proposed by Squires et al^5^ should then try to integrate the dynamic nature of the context that interacts with, and is transformed by the characteristics of the intervention being implemented. As highlighted in the findings “*informants frequently discussed multiple attributes and features simultaneously, illustrating the complexity and interrelatedness of context*” (5; p. 4). In this respect, it seems essential to explore how to account for the nonlinearity of the processes and the interconnection between different contextual factors in describing the context of a KT intervention.

## Embracing Context Diversity

 Apart from the specific physical context in which an intervention is implemented, the concept of context should also encompass the macro level (socio-economic or epidemiological), and meso level (institutional) contexts that are known to considerably influence the uptake and impact of an intervention.^1^

 In the Squires et al study,^5^ the qualitative material that served as the basis to develop the list of contextual factors came only from stakeholders in high-income countries. A growing number of implementation studies take place in low- and middle-income countries (LMICs) and it would be important to ensure that the reality of their context is also captured in the list of contextual attributes and features.

 The problem of reporting contextual factors is also present in the KT/IS literature from LMICs. Alonge et al^13^ reviewed published implementation studies in LMICs and noted a lack of consistent reporting of implementation characteristics such as the description of the context and the intervention itself. A question that deserves further investigation is whether the list proposed by Squires and colleagues would be relevant in identifying contextual influences on the success or failure of intervention implementation in LMICs.

 There is some evidence supporting that such list would probably need to be adapted for its application in LMICs. The study by Bergstorm et al^14^ assessed the relevance of the organizational context cornerstone of the PARiHS framework and whether additional organizational factors influenced implementation of maternal and child health interventions in Uganda. Their findings support the fact that the PARiHS framework is relevant for LMICs, but they also identified further contextual factors that influenced KT in this specific context. Indeed, factors such as commitment, informal payment, and community involvement were found to influence the implementation of interventions. It is also important to provide adapted measurement tools to capture contextual factors relevant for implementation in LMICs. One such tool is the Context Assessment for Community Health tool that has been developed based on the PARiSH framework^7^ and validated in several languages.^15^

## Conclusion

 Acknowledging that context is inextricably linked to the implementation of healthcare interventions would lead to a better consideration of this aspect in KT studies. Squires et al reinforce the importance of considering the multiple contextual elements that can influence the successful implementation of healthcare interventions. This study also provides much needed conceptual clarity to better understand the role of context in KT/IS, which is essential for developing common assessment tools to capture it.

 Reporting on the implementation context is important not only to ensure the fidelity and replicability of interventions, but also to consider the conditions and factors that may have influenced the implementation of the intervention, its process and its results. Using a common terminology to document contextual factors would improve transparency in implantation research and facilitate comparison between studies.

 In summary, the Squires et al paper provides a very comprehensive, helpful and robust basis to develop a framework and a measurement tool to assess contextual factors in a variety of implementation studies. More extensive validation of its robustness and comprehensiveness could be carried out, notably in LMICs.

## Ethical issues

 Not applicable.

## Competing interests

 Author declares that she has no competing interests.

## Author’s contribution

 MPG is the single author of the paper.
